# Fractured Full-Arch Tooth-Supported Zirconia Bridge: Thin Design, Surface Damage, and Excessive Cement Layer Thickness

**DOI:** 10.3390/reports9010049

**Published:** 2026-02-02

**Authors:** João Paulo Mendes Tribst, Bart Jansen, Rafaela Oliveira Pilecco, János Kodolányi, Amanda Maria de Oliveira Dal Piva

**Affiliations:** 1 Department of Reconstructive Oral Care, Academic Centre for Dentistry Amsterdam (ACTA), Universiteit van Amsterdam and Vrije Universiteit, Gustav Mahlerlaan 3004, 1081 LA Amsterdam, Noord-Holland, The Netherlands; j.p.mendes.tribst@acta.nl (J.P.M.T.); b.j.jansen@acta.nl (B.J.); 2 Department of Conservative Dentistry, Faculty of Dentistry, Federal University of Rio Grande do Sul (UFRGS), R. Ramiro Barcelos, 2492, Porto Alegre 90035003, RS, Brazil; rafaela-pilecco@hotmail.com; 3 Department of Dental Materials Science, Academic Centre for Dentistry Amsterdam (ACTA), Universiteit van Amsterdam and Vrije Universiteit, Gustav Mahlerlaan 3004, 1081 LA Amsterdam, Noord-Holland, The Netherlands; j.kodolanyi@acta.nl

**Keywords:** dental prosthesis, dental restoration failure, resin-bonded bridge

## Abstract

Zirconia is widely used in full-arch restorations due to its strength and aesthetics, but failures can still affect its performance in clinical practice. In this report, a full-arch tooth-supported zirconia bridge fractured prematurely (eleven months), encouraging an investigation into its design and failure mechanisms. STL files obtained from the dental laboratory revealed regions of reduced framework thickness, falling below the manufacturer’s recommendations. Fractographic analysis of the fractured pieces indicated a multifactorial failure pattern. Notable features included a thick cement layer, surface damage likely caused by the CAM bur during milling, and occlusal wear affecting the glazed surface. Crack propagation was observed in an occlusal-to-cervical direction. While no single factor could be definitively identified as the primary cause, the failure is attributed to the combined effect of insufficient design, surface damage, and biomechanical overload. Importantly, most such factors are not visible before failure, raising questions about the proper evaluation of zirconia-based restorations prior to their cementation.

The present article is an interesting image modality. The aim is not to present the full case but rather to report a failed condition and present some of the design aspects and fracture features that can be observed and related to a possible cause.

Fixed dental prostheses have long been a crucial alternative in the rehabilitation of edentulous and partially edentulous patients, providing functional restoration, aesthetic improvement, and enhanced quality of life [1,2]. Among the various materials available for full-arch restorations, zirconia has gained increasing popularity due to its superior mechanical properties, excellent biocompatibility, and favorable esthetic outcomes. In summary, the development of monolithic zirconia bridges has significantly advanced the dental field, overcoming problems related to chipping of veneering porcelain and offering improved long-term performance [3].

Numerous studies have reported high survival rates and patient satisfaction associated with zirconia restorations [2,4]. However, despite their strength and longevity, zirconia prostheses are not exempt from complications. Biological and mechanical failures, such as fracture [3,5], and, in some cases, caries in abutment teeth, can compromise the treatment success [6]. Zirconia’s high stiffness, while beneficial, may also increase the risk of catastrophic failure under certain clinical conditions, such as fatigue.

Zirconia framework failure is a multifactorial phenomenon influenced by material properties, prosthetic design, manufacturing processes, and clinical parameters [7,8,9]. Inadequate framework design, insufficient connector dimensions, and surface damage have been shown to significantly increase the risk of fracture under chewing loading [10]. Additionally, low-temperature degradation of yttria-stabilized tetragonal zirconia polycrystal (Y-TZP), characterized by phase transformation from tetragonal to monoclinic, can compromise mechanical strength over time, particularly in moist environments such as the oral cavity. In addition, veneering ceramic chipping and interfacial delamination may also contribute indirectly to framework failure by altering stress transmission. Furthermore, flaws introduced during CAD/CAM milling, sintering, or surface treatments such as aggressive grinding and sandblasting, can act as crack initiation sites. Occlusal overload, parafunctional habits, and improper cementation techniques further exacerbate these risks. Recognizing and understanding these factors is essential for appropriately contextualizing and interpreting cases of zirconia framework failure [11,12,13].

This image compendium presents a fractured zirconia bridge collected from the clinic from a specialization program in prosthodontics and implant dentistry. The bridge had a long span from canine to canine (abutments 27, 26, 25, 24, 14, 15, 17) and the antagonist arch presented intact natural teeth (teeth 34–43), one monolithic zirconia crown (tooth 44), and two implant-supported monolithic zirconia bridges (teeth 45–47 and 35–37). It was reported that a provisional bridge was fabricated using PMMA milled material and placed intraorally to evaluate occlusion, phonetics, and aesthetics. Upon approval, definitive impressions were taken, and a monolithic zirconia (priti^®^multidisc multicolor, Sea Dental, Germany) bridge was fabricated. In contact with the laboratory, a minimum framework wall thickness of 0.8–1.0 mm was used in axial and occlusal areas. Connector dimensions were designed with a minimum cross-sectional area of 9–12 mm^2^. The prosthesis was designed in centric relation and customized to the patient’s aesthetic requirements. The final restoration, as received from the dental lab, can be seen in Figure 1. The abutments were cleaned to remove temporary cement residues and subsequently polished. The prosthesis was ultrasonically cleaned and sandblasted (50 μm Aluminum oxide), followed by external polishing of the margins using Katana spirals. Cementation was performed with Fuji Plus (GC), resulting in an accurate seating. After setting, the excess cement was carefully removed, and the cleanability of the restoration was verified. All margins were refined with polishing paste and a rubber cup. Occlusion and articulation were considered satisfactory based on a group function occlusal scheme appropriate for a full-arch fixed bridge, with distributed occlusal contacts in maximum intercuspation and controlled guidance during lateral and protrusive mandibular movements. Static and dynamic occlusal contacts were evaluated clinically using articulating paper and shimstock foil, with particular attention paid to the elimination of premature contacts and non-working side interferences.

It is crucial to ensure that the zirconia framework maintains adequate thickness throughout the fabrication and clinical adjustment processes to preserve its mechanical strength. In this case, according to the dentist responsible for the case, no intentional adjustments were made to the zirconia bridge framework before cementation that would compromise its structural integrity. The clinical protocol involved initially placing the restorations made of PMMA and composite materials, and any minor occlusal modifications were performed primarily on these provisional structures or antagonists rather than on the definitive zirconia restoration. Careful attention was given to avoid grinding or thinning of the zirconia framework, particularly in critical areas such as connectors and abutments. The absence of visible adjustment marks on the framework supports this approach. Despite that, we must highlight the importance of verifying framework thickness before and after any adjustment, as excessive reduction may increase the risk of framework failure.

After 19 months post-placement, the patient returned with complaints of mobility in the prosthesis. The patient reported that while chewing a soft bread, a sudden “crack” sound was heard and perceived, in the cervical region beneath the fixed dental prosthesis, a sensation of displacement, as if the restoration had seated or rested more firmly on the adjacent tooth. Figure 2 and Figure 3 present the failed prosthesis.

Clinical examination confirmed that the bridge had fractured into two separate segments (between 14 and 15, and between 24 and 25) and was no longer repairable intraorally. Consequently, the decision was made to remove the prosthesis and restart the treatment.

To better understand the cause of the fracture, the dental laboratory was contacted, and the original STL file of the bridge was requested for analysis (Figure 4). The digital file was imported into a Computer-Aided Design (CAD) software (Rhinoceros, Version 5.0, Robert McNeel & Associates, 2012), where a detailed inspection of the bridge design was conducted. Perpendicular cross-sectional cut planes were created through each abutment tooth to evaluate the preparation geometry and thickness. This simple analysis aimed to assess the form and uniformity of the prosthesis, particularly at fracture sites (Figure 5). Regions with thicknesses less than 1.2 mm and non-uniform outlines can be observed. Based solely on this figure, it is unclear whether the tooth preparation was insufficient or if the minimum thickness requirement was not enforced during the CAD process.

After removal of the fractured monolithic zirconia bridge, a fractographic analysis was conducted on both separated segments to investigate the nature of the failure. During removal, the right segment detached spontaneously, preserving the fracture surface (pontic between 14 and 15), while the left segment (pontic between 24 and 25) remained firmly attached and was therefore sectioned (element 24 was cut in half) using a rotary diamond bur. The objective was to evaluate the fracture features of both fractures to identify the possible origin.

Both surfaces were cleaned and examined under an optical microscope and scanning electron microscopy (EVO LS15; Carl Zeiss, Oberkochen, Germany), and documented photographically (Figure 6 and Figure 7). Images were recorded at different magnifications, ranging from 172× to 500× (Figure 6) and from 161× to 500× (Figure 7). The direction of crack propagation and surface textures were analyzed to provide detailed insight into the failure mechanism of the zirconia bridge. We also check the cement layer thickness and surface damage. The thickness of the cement layer was not intentionally evaluated as part of the SEM. Instead, it was incidentally observed during the imaging examination performed to analyze the fractured zirconia framework. This finding was documented qualitatively as part of the fracture assessment. Unfortunately, the cement layer was not entirely uniform across all abutments. Consequently, the cement thickness could only be reliably measured on one abutment during the imaging examination. Therefore, this assessment is a localized observation rather than a comprehensive evaluation.

The fracture between elements 14 and 15 (Figure 6) originated in the occlusal area of the 15 (close to the connector). Two fracture events are observed, corresponding to defects located near the contact areas. These flaws appear to have acted as crack origins or deviation sites, contributing to accelerated crack propagation and failure. Additionally, no glaze layer is visible, suggesting its removal during occlusal adjustment or wear. In the mid-region opposite to the origin, a well-defined compression curl is visible, characteristic of the terminal compression zone commonly observed in brittle materials [7,8]. This indicates that the fracture was arrested in this region due to compressive collapse after critical crack propagation [7]. In addition, the specimen exhibits typical features of a defect located in the cervical region (area highlighted in green). In this area, a superficial flaw with morphology consistent with diamond bur-induced damage can be observed, indicating a secondary event.

In Figure 7, the fractographic analysis illustrates the failure between elements 24 and 25, which occurred in the occlusal surface (glaze) of the connector region. The area highlighted in red shows a lateral defect (palatinal), likely introduced by rotary instrumentation during preparation or finishing. Although such defects can act as fracture origins, in this case, the lateral defect does not appear to be the primary origin due to the absence of characteristic crack initiation features [8]. Conversely, the occlusal defect (highlighted in green) exhibits a rough, irregular morphology with microvoids, making it more indicative of the fracture origin. These features suggest localized stress concentration and pre-existing damage accumulation within the microvoids before crack propagation.

The cement layer thickness was also explored (Figure 8). The representative images show a heterogeneous cement layer with variable thickness across different areas of the crown, ranging from 106 to 440 µm. In addition, air bubbles of various sizes were observed throughout the resin-modified glass ionomer cement.

This case highlights the importance of patient follow-up, precise CAD, and consideration of biomechanical stress distribution when planning and maintaining full-arch zirconia restorations. It is important to emphasize that prevention remains the most effective and cost-efficient strategy to avoid ceramic fractures. This includes performing proper polishing of the pieces during post-processing and after adjustments, thereby reducing crack initiation [7,8,9,10,11]. Despite having access to the STL file, clinical examination, and fractographic analysis, it was not possible to identify a single predominant factor responsible for the premature failure of the restoration. The most plausible explanation is that a combination of controllable factors, such as uniform ceramic thickness, a consistent cement layer, and proper surface finishing and occlusal adjustments, was overlooked. Additionally, the use of adhesive resin cements over conventional glass ionomer cements can be an option to enhance bonding strength. Clinically, the challenge lies in the fact that several contributing factors are difficult to detect and address before a catastrophic failure occurs.

## Figures and Tables

**Figure 1:**
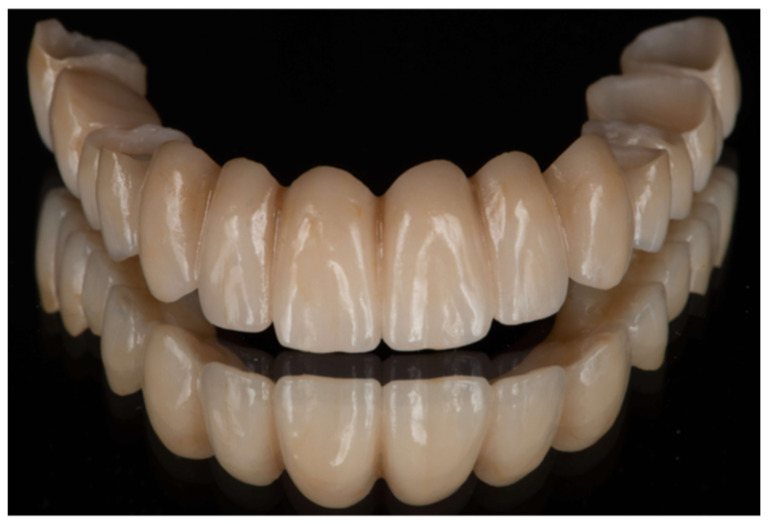
Final prosthesis made of monolithic zirconia.

**Figure 2:**
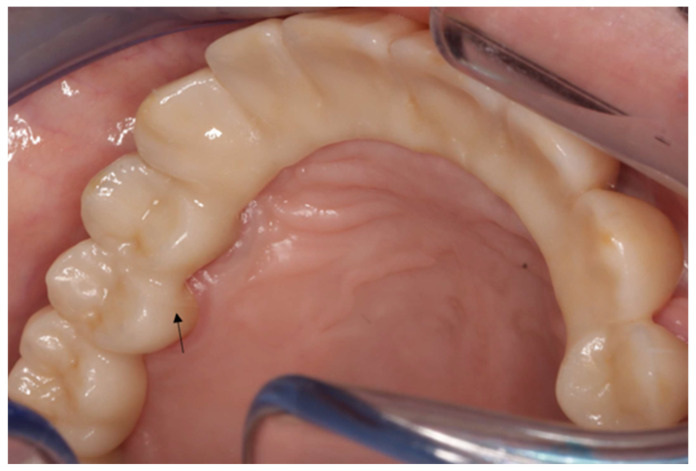
Intraoral view of the zirconia superstructure. A barely visible crack between teeth 14 and 15 was detected.

**Figure 3:**
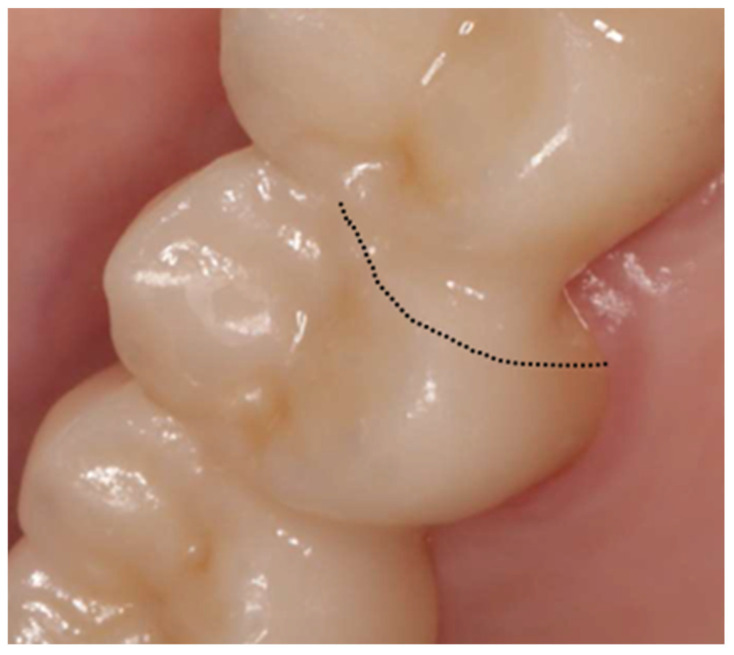
For didactic purposes, the fracture line is outlined in black.

**Figure 4:**
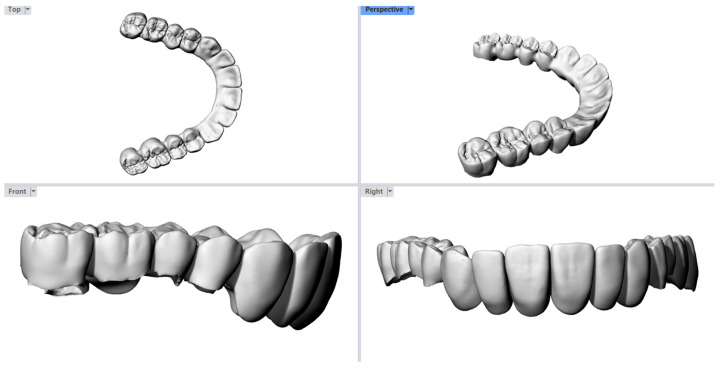
The STL file of the bridge was requested from the lab. They delivered it according to the final design that was milled for the patient. Clinically, there are no visual issues or notable concerns in the external design that could have prevented the treatment from moving forward.

**Figure 5:**
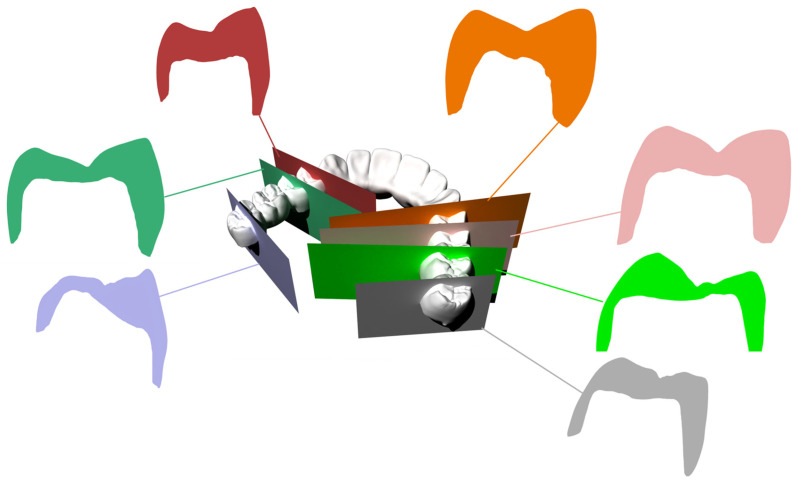
The STL file was exported to Rhinoceros CAD software. Using the CutPlane command, several inspection paths were created for each abutment tooth (one in each color) based on the cross-sections of the thinnest areas (min 0.8 mm).

**Figure 6:**
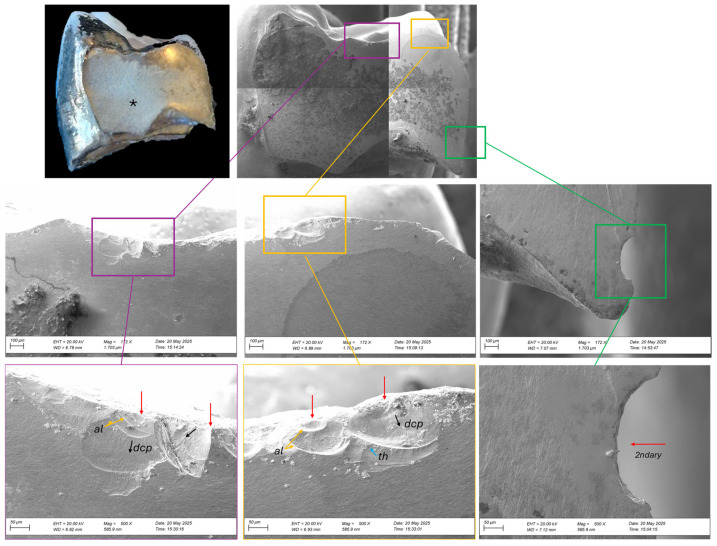
Fractographic analysis of the failure origin in the defect between elements 14 and 15. The overview image shows the entire fracture surface with colored boxes indicating regions of interest. In the cervical area (highlighted in green), a surface defect consistent with damage caused by rotary instrumentation—likely from a diamond bur—is identified as a secondary event. In the occlusal surface, two fracture events are observed at defect sites adjacent to the contact points, acting as crack initiation sites (red arrows). From these sites, crack propagation features (direction of crack propagation—dcp, arrest line—al, and twist hackle—th) can be followed toward the opposite side, terminating in a compressive region characterized by a well-defined compression curl (*).

**Figure 7:**
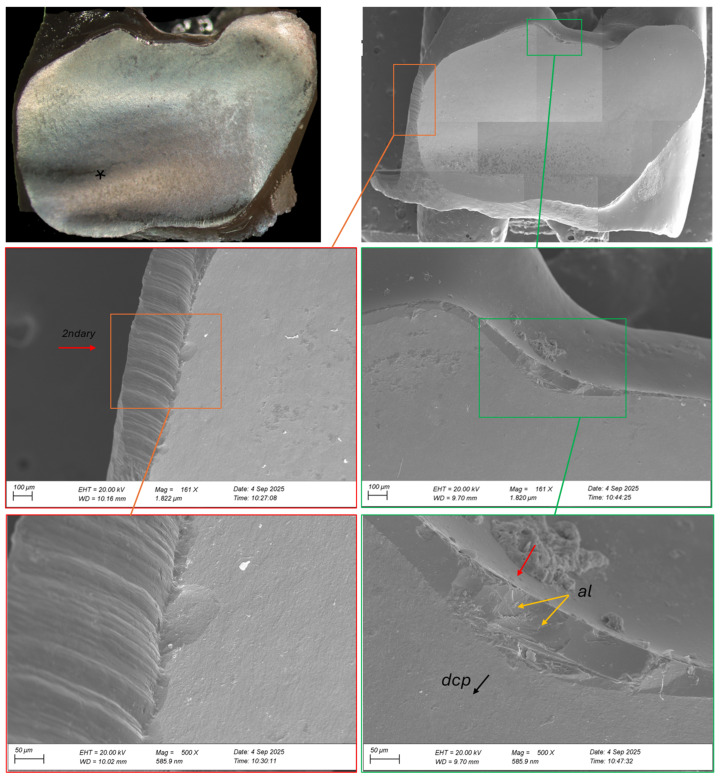
Fractographic analysis of the fracture origin between elements 24 and 25. In the occlusal area (green), a rough defect in the glaze with irregular morphology and microvoids marks the primary fracture origin. From this site, the crack propagated (direction of crack propagation—dcp, and arrest line—al) toward the cervical region, terminating in a compression curl (*). In the palatal area (red), a surface consistent with rotary instrumentation damage is observed, acting as a secondary fracture site.

**Figure 8:**
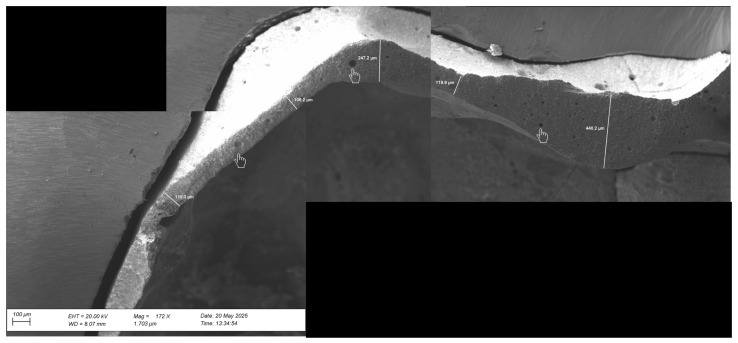
Scanning electron microscopy analysis of the cement layer thickness (magnification of 172×). The indicators (hand-shaped pointer) show air bubbles of various sizes throughout the resin-modified glass ionomer cement. As well as the heterogeneous cement layer thickness (measurement lines).

## Data Availability

The original contributions presented in this study are included in the article. Further inquiries can be directed to the corresponding author.
